# LDH-Indomethacin Nanoparticles Antitumoral Action: A Possible Coadjuvant Drug for Cancer Therapy

**DOI:** 10.3390/molecules29143353

**Published:** 2024-07-17

**Authors:** Kelly Costa Alves, Carlos Emmerson Ferreira da Costa, Cláudio Márcio Rocha Remédios, Danielle Queiroz Calcagno, Marcelo de Oliveira Lima, José Rogério A. Silva, Cláudio Nahum Alves

**Affiliations:** 1 Programa de Pós-Graduação em Ciência e Engenharia de Materiais, Universidade Federal do Pará, Belém 66075-110, Brazil; 2 Programa de Pós-Graduação em Química, Universidade Federal do Pará, Belém 66075-110, Brazil; 3 Instituto de Ciências Exatas e Naturais, Universidade Federal do Pará, Belém 60740-000, Brazil; 4 Núcleo de Pesquisas em Oncologia, Hospital Universitário João de Barros Barreto, Universidade Federal do Pará, Belém 66075-110, Brazil; 5 Programa de Pós-Graduação em Ciências e Meio Ambiente, Universidade Federal do Pará, Belém 66075-110, Brazil; marcelolima622@gmail.com; 6 Computer Modeling of Molecular Biosystems (CompMBio), Universidade Federal do Pará, Belém 66075-110, Brazil; 7 Catalysis and Peptide Research Unit, University of KwaZulu-Natal, Durban 4041, South Africa

**Keywords:** indomethacin, layered double hydroxides, coprecipitation, MTT, chemotherapeutic treatment

## Abstract

Indomethacin (INDO) has a mechanism of action based on inhibiting fatty acids cyclooxygenase activity within the inflammation process. The action mechanism could be correlated with possible anticancer activity, but its high toxicity in normal tissues has made therapy difficult. By the coprecipitation method, the drug carried in a layered double hydroxides (LDH) hybrid matrix would reduce its undesired effects by promoting chemotherapeutic redirection. Therefore, different samples containing INDO intercalated in LDH were synthesized at temperatures of 50, 70, and 90 °C and synthesis times of 8, 16, 24, and 48 h, seeking the best structural organization. X-ray diffraction (XRD), vibrational Fourier transform infrared spectroscopy (FT-IR), scanning electron microscopy (SEM), spectrophotometric analysis in UV-VIS, and differential thermogravimetric analysis (TGA/DTA) were used for characterization. Our results indicate that higher temperatures and longer synthesis time through coprecipitation reduce the possibility of INDO intercalation. However, it was possible to establish a time of 16 h and a temperature of 50 °C as the best conditions for intercalation. In vitro results confirmed the cell viability potential and anticancer activity in the LDH-INDO sample (16 h and 50 °C) for gastric cancer (AGP01, ACP02, and ACP03), breast cancer (MDA-MB-231 and MCF-7), melanoma (SK-MEL-19), lung fibroblast (MRC-5), and non-neoplastic gastric tissue (MN01) by MTT assay. Cell proliferation was inhibited, demonstrating higher and lower toxicity against MDA-MB-231 and SK-MEL-19. Thus, a clinical redirection of INDO is suggested as an integral and adjunctive anticancer medication in chemotherapy treatment.

## 1. Introduction

Cancer is one of the world’s biggest public health concerns, despite considerable research advances in recent years. Global estimates indicate a gradual increase in cancer case number and associated deaths, and each year, more than 14 million people are diagnosed with cancer [1]. In the past decade, there has been a 20% increase in incidence, and it is projected that by 2030, over 25 million new cases will occur. Notably, in 2023, in Brazil, an estimated 704,000 new cancer cases were expected [2].

Recent nanotechnology developments have enabled scientists to develop potential innovations in auxiliary treatment and have contributed to the search for potential innovations in cancer chemotherapy, reducing side effects by developing nanoparticulate materials capable of carrying new therapeutic drugs in controlled release systems [3]. Among these materials, inorganic ceramics, such as layered double hydroxides (LDH), are characterized by nanocomposites containing positively charged layers of mixed-valence hydroxides comprising di and trivalent metal ions, with hydrated anions intercalated in the interlamellar domain [4]. These properties define the absorption capacity of organic species in the anionic form and their efficiency in transporting molecules for pharmaceutical purposes, making their administration in the organism possible [5]. Hydrotalcite-type LDHs are increasingly used as a hybrid matrix that binds molecules with pharmacological activity, aiming at their protection and stability, and consequently avoiding their undesirable rapid and uncontrolled degradation [3,6].

Recently, the therapeutic targets studied have included non-steroidal anti-inflammatory drugs (NSAIDs) such as indomethacin (INDO), derived from indoleacetic acid, or [1-(4-chlorobenzoyl)-5-methoxy-2-methylindol-3-yl] acetic acid [7]. Its activity is based on the inhibition of the enzyme cyclooxygenase (COX) and neutrophil activity reduction relating to two common isoforms: cyclooxygenase-1 and cyclooxygenase-2 (COX-1 and COX-2, respectively) [8]. COX-1 is an enzyme that is engaged in the body’s homeostasis. COX-2 is primarily responsible for the prostanoid mediators of inflammation. Both present with structurally similar isoforms at their active sites, forming a hydrophobic channel where substrates such as arachidonic acid, a key mediator of inflammation, catalyzes to the prostaglandin H2 (PGH2) endoperoxide in dioxygenase activity and subsequently to peroxidase, catalyzed by the two COX isoforms [8,9].

The relationship with NSAIDs is based on anticancer activity by the inhibition of COX-2 [10]. Studies have shown that prostaglandins generated by COX-2 are responsible for invasiveness, angiogenesis, and tumor progression [11,12]. Thus, inhibition of COX-2 could disrupt carcinogenesis by preventing cancer development and leading to the regression of the developed tumor [8]. However, due to its inhibitory effect on COXs, INDO has significant adverse effects, which are dose-dependent. Consequently, it has been restricted to therapeutic alternatives that are not effective [13].

Furthermore, under prolonged administration, it can lead to very severe gastrointestinal disorders, such as ulcerations and hemorrhages, [14] and renal complications [15]. COX-1 has been highlighted as a dominant isoform in gastric epithelial cells and as the primary source of cytoprotective prostaglandins. Thus, inhibition of COX-1 could cause adverse gastric events, such as severe ones observed in steroidal anti-inflammatory drugs (SAIDs) treatment [8,16,17]. Similarly, inhibition of the renal protective effects of prostaglandins as vasoconstrictors could aggravate pre-existing renal dysfunction, causing renal failure [15].

Therefore, to reduce the side effects of INDO, it has been proposed that this drug be introduced into carrier systems [18,19,20]. Thus, developing strategies that modify INDO to increase its efficiency and decrease its toxicity could lead to the clinical redirection of this drug as a potential anticancer target. In this study, the best time and temperature conditions for producing LDH-INDO intercalated nanocomposites were optimized. In sequence, cytotoxic potential, cell viability, and anticancer activity using the drug alone and within the chosen intercalation (LDH-INDO) were also evaluated.

## 2. Results

### 2.1. X-ray Diffraction

**Figure 1:**
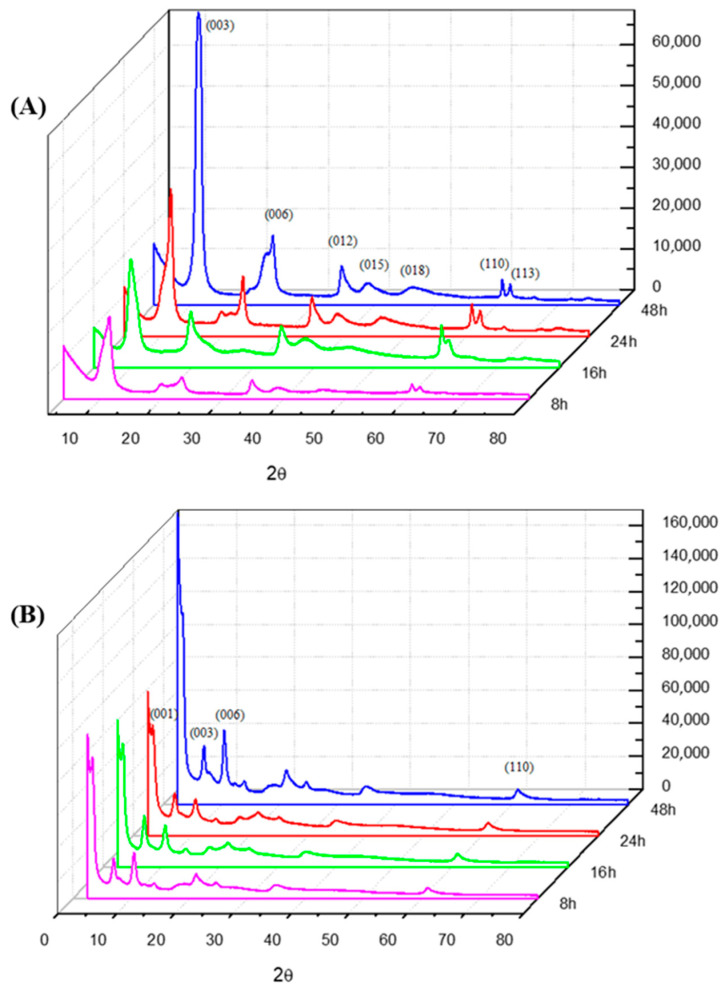
Diffractogram plots for (**A**) LDH and (**B**) LDH-INDO systems at 50 °C over the course of 8, 16, 24 and 48 h, respectively.

The X-ray diffraction results show the samples of pure LDH, prepared at different aging times (8, 16, 24, and 48 h) and temperatures (50, 70, and 90 °C), as shown in Figure 1A. During a coprecipitation rotation, the increase in the aging time of the particles is suitable for a rise in flat crystallinity (003) (Figure 1A). Thus, the results in Figure 1A show that LDH–48 h–50 °C presented better quality, an example of LDH-16 h-50 °C presenting more stable structural integrity.

Table 1 presents the data for the interplanar distances (dhkl), Miller indices (hkl), 2θ values, the crystallite size (T), and layer stacking number of each LDH sample (REFs). These samples were prepared at various time intervals (8, 16, 24, and 48 h) and temperatures (50, 70, and 90 °C). In addition, Figure 1B illustrates the diffractograms of LDH-INDO intercalated samples prepared at aging times of 8, 16, 24, and 48 h at a temperature of 50 °C.

These results are more evident in Table 2, where data on interplanar distances (dhkl), Miller indices (hkl), 2θ values, the crystallite size (T) and stacking number of the layers are displayed for LDH-INDO samples prepared over different time intervals (8, 16, 24, and 48 h) and temperatures (50, 70, and 90 °C), respectively.

As for the crystallographic parameters described in Table 3, corresponding to the symmetry of unit cells “a” and “c”, there is a slight variation in the interplanar distances characterizing the material synthesized as polytype 3R, a hexagonal unit cell, with an interlamellar distance (d003) equal to C/3, as described in the literature [21].

### 2.2. FT-IR Vibrational Spectroscopy, Scanning Electron Microscopy (SEM) and Thermogravimetric Analyzes (TGA/DTA)

**Figure 2:**
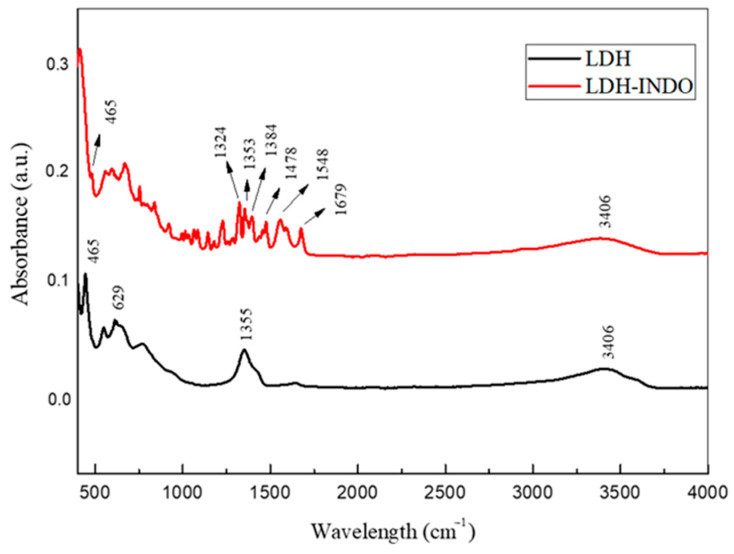
FT-IR plots for LDH and LDH-INDO systems at 50 °C and 16 h.

In the spectra shown in Figure 2, broad bands in the 3400 cm^−1^ range are observed, which is characteristic of the stretching of the OH group of hydration water molecules, probably present in the lamellae [21,22]. The sample interspersed with INDO showed several absorption bands characteristic of the drug, registering bands in the range of 1345–1385 cm^−1^, corresponding to the permanence of O-M-O metals and possibly some nitrate anions still present in the structure.

**Figure 3:**
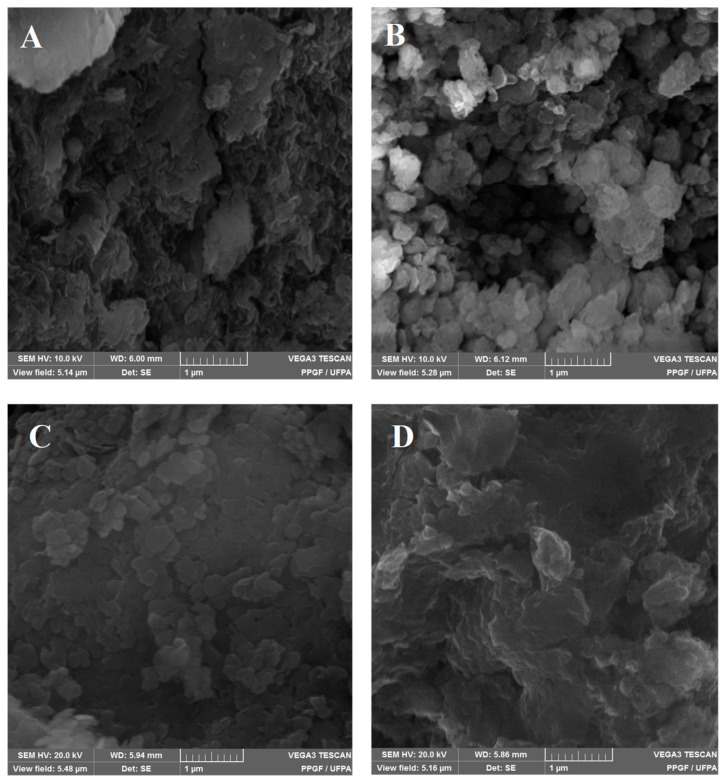
Micrographs of LDH samples at (**A**) 16 h, 50 °C, and (**B**) 16 h, 90 °C. Interleaved LDH-INDO samples at (**C**) 16 h, 50 °C and (**D**) 16 h, 90 °C.

The micrographs of the LDH and LDH-INDO samples, prepared for 16 h at a temperature of 50 °C, are shown in Figure 3. Figure 3A,B show the micrographs for the LDH samples, and Figure 3C,D show the LDH-INDO samples.

**Figure 4:**
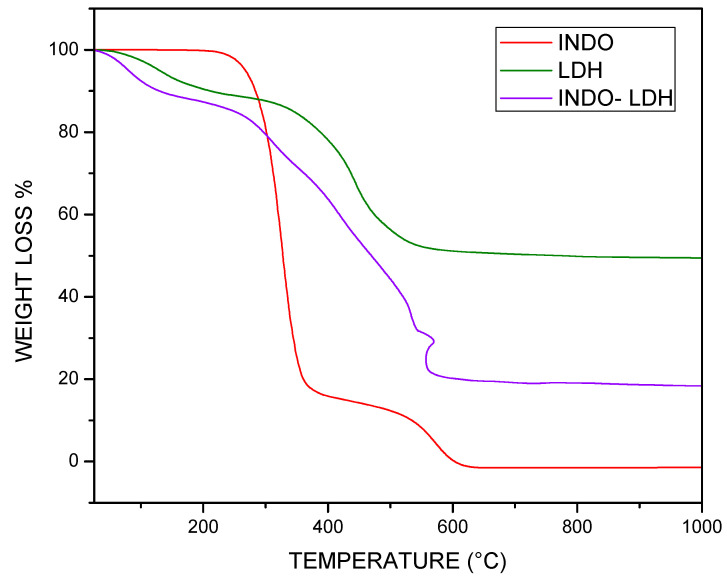
TGA curves of samples of LDH-INDO-16 h-50 °C, LDH-16 h-50 °C, and INDO.

The thermograms in Figure 4 illustrate the TGA curves of the isolated INDO, LDH, and LDH-INDO samples, prepared at 16 h time intervals at a temperature of 50 °C, per coprecipitation route. In this illustration, for the LDH sample (purple color), the first mass loss of 11.16% starts at just before 200 °C, possibly related to the removal of poorly adsorbed water due to the low temperature at which it occurred. The second mass loss at 400 °C, 38.21%, is related to water hydroxyl groups (dehydroxylation). This comes from the adsorbed interlayer and the decomposition of intercalated anions.

**Figure 5:**
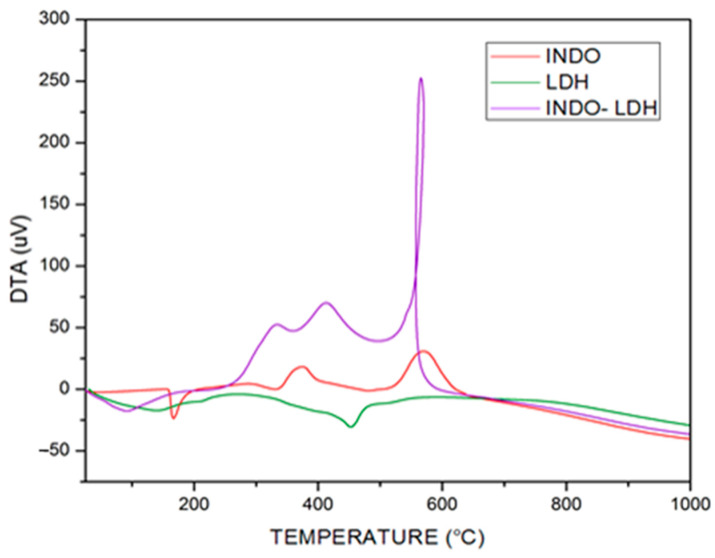
DTA curves of indomethacin samples, LDH-INDO-16 h-50 °C and LDH-16 h-50 °C.

The thermal analysis derivative (DTA) curve (Figure 5) defines the maximum and minimum points representing the limits of the decomposition steps, which indicate the beginning and end of the thermal events of the mass loss.

### 2.3. Cell Viability Assay

The viability of the cells after exposure and optimal concentrations for treatment were evaluated by MTT cytotoxicity assay (shown in Table 4) using pure INDO and interleaved LDH (Mg^2+^/Al^3+^) at a time interval of 16 h and a temperature of 50 °C, given that this was the best result obtained from the characterizations presented above.

For isolated INDO, a mean inhibitory concentration with 50% activity (IC50) at a time of 72 h in the tested cell lines was 0.94 mM, with a standard variation of 0.39 mM (95% CI 0.33–0.46) to 1.55 mM (95% CI 1.26–1.92). Cell proliferation was inhibited in all studied cell lines, with more cytotoxicity observed in MDA-MB-231 and less in SK-MEL-19. On the other hand, the gastric cancer lineages presented a mean IC50 of 0.97 mM, showing higher and lower cytotoxicity against AGP01 and ACP02, respectively. Isolated INDO showed higher cytotoxicity in breast cancer cell lines, with a medium IC50 of 0.45 mM. However, in standard cell lines (MN01 and MRC5), the IC50 average was only 1.06 mM, which would make the use of INDO in isolation possible in gastric cancer (except in cases where ascites fluid has formed) and melanoma.

For LDH-INDO intercalation, the mean IC50 at 72 h in the tested cell lines was 2.58 mM, ranging from 0.89 mM (95% CI 0.81–0.96) to 3.79 mM (95% CI 2.95–4.87). INDO-LDH inhibited the cell proliferation of all the studied strains, and was also more cytotoxic to MDA-MB-231 and less cytotoxic to SK-MEL-19. In gastric cancer, the mean was 2.78 mM, demonstrating, this time, higher and lower cytotoxicity against AGP01 and ACP03, respectively. Breast cancer cell lines showed higher cytotoxicity with a medium IC50 of 1.30 mM. In standard cell lines, the average IC50 was only 2.95 mM, increasing the feasibility of using the intercalated LDH matrix for treatment, except for in intestinal-type gastric cancer and melanoma.

It was observed that, for LDH-INDO, there was an overall decrease in cytotoxicity, and an increase in IC50. The results show that the entrainment of LDH matrices decreases their toxicity against normal tissues. It was shown that intercalated molecules need to be about three times larger to demonstrate some cytotoxicity, opening the possibility for clinical redirection drugs as an anticancer therapy. However, no studies have been found in the literature demonstrating the biological effects of LDH-INDO with anticancer activity, reinforcing the importance of ongoing studies.

**Figure 6:**
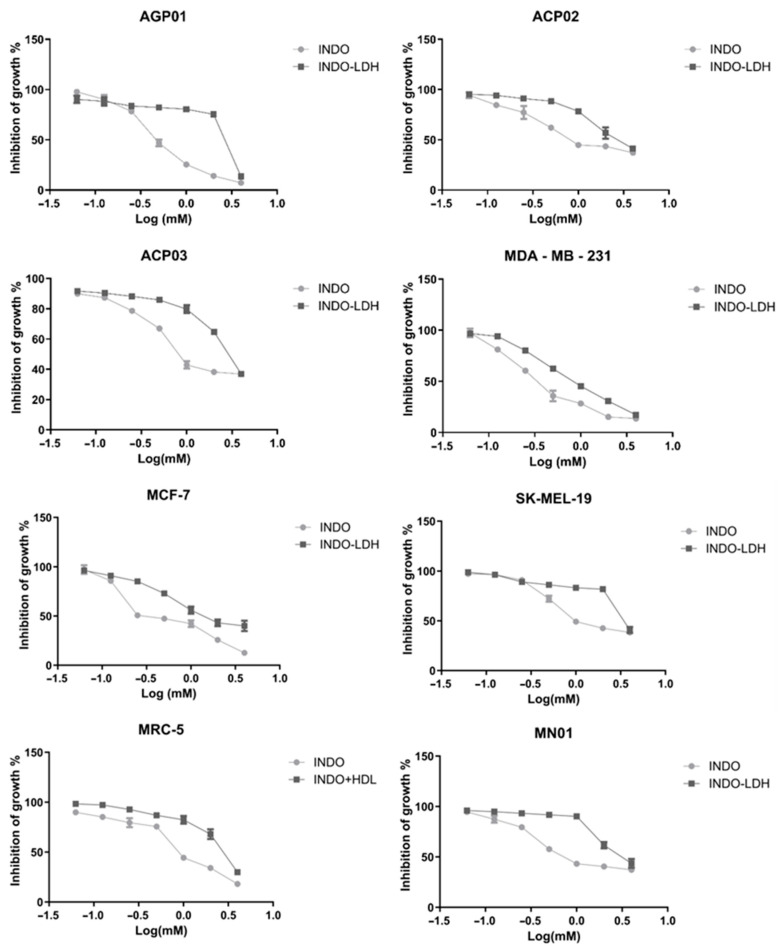
Cell growth inhibition (in percent) by logarithmic concentrations used in the studied cell line.

The graphs below (Figure 6) show the curve of cell growth inhibition (as a percentage) by the log of the concentrations used in the studied cell lines. The marked drop in cell growth observed for LDH-INDO may indicate that 72 h is when drug release from the matrix leads to its peak activity, which needs to be confirmed by functional cytotoxicity time assays. A statistically significant difference (*p* < 0.05) was observed by lineage for the INDO vs. LDH-INDO. These results confirm that LDH-INDO has different activity than INDO alone, and the predominant factor for this diversity seems to be the protective effect of LDH intercalation.

## 3. Discussion

In Figure 1A, the basal plane d(003) increases as the temperature increases. However, the values obtained for these planes do not suffer significant differences when correlated with the increase in the aging time of the particles used during syntheses. It is also observed that plane d(110) remains practically unchanged as a function of temperature and time, with differences in the values of 2θ being evidenced, which could be related to the crystallinity profiles obtained for each sample. Significant differences were found in the sizes of the LDH particles and the number of layers as the synthesis temperature and the aging time increased. The diffractograms shown in Figure 1B are relative to LDH-INDO intercalated samples, prepared at aging times of 8, 16, 24, and 48 h and at a temperature of 50 °C. It is possible to observe the continuity of planes 003, 006, and 110 relating to the LDH formed and the appearance of plane 001 relating to the intercalated drug indomethacin. However, the same does not occur in LDH samples between 16 and 72 h at temperatures below 50 °C, where the refracted planes (Figure S1) show two possible peaks in all regions of low angles (10 < 15°).

Regarding the intercalations, it is possible to observe the presence of the plane (001), which corresponds to the presence of the drug. However, the crystallinity in the plane (003) is low compared to the plane (006), showing probable structural disorganization. As early as 16 h, an increase in (001) and (003) and a decrease in the plane (006) occurs, emphasizing the effective intercalation of INDO and the structural organization of the formed material. From 16 h of synthesis, a decrease in the plane referring to INDO and the plane (003) is observed, as well as an increase in the plane (006), suggesting a decrease in the intercalation, as well as the onset of structural malformation. These results are more evident in Table 2, showing the basal spacings related to INDO increase as the temperature increases over 8 h of prepared syntheses. At 16 h, it is possible to observe an even more significant increase in plane (001) spacing. However, there was relative stability when correlated with an increase in temperature. Since a significant decrease in d(001) and an increase in d(003) occurs at 16 h, suggesting a possible maximum limit of intercalation at a synthesis time of 16 h at 50 °C. These results demonstrate an unprecedented finding in the literature. To the best of our knowledge, there are no studies that demonstrate in XRD the maximum limit of intercalation of INDO in hybrid matrices of magnesium and aluminum (Mg-Al-LDH), nor is there any specific study that proves the direct influence of temperature and time under the synthetic route of intercalation (LDH-INDO) by coprecipitation [23,24,25]. It was also observed when analyzing the intercalations that an increase in the aging temperature in the synthesis favors reducing the number of stacking lamellar layers. Consequently, this establishes a direct relationship between the aging temperature and a decrease in the size of the particles of the formed materials.

The crystallographic parameters are described in Table 3. The network/grid parameter a, which corresponds to the average distance of the cations present within the layers, presents values of approximately 3 Å throughout all of the samples, remaining practically unchanged for both pure LDH and the intercalated hybrids. This finding reveals the probable tendency of crystalline network uniformity in interlamellar formation. Parameter c, linked to the layer thickness and interlayer distance, exhibits variations in all of the LDH and LDH-INDO samples. These variations in interplanar distances suggest materials synthesized as polytype 3R, a hexagonal unit cell with interlamellar distance d003 equal to C/3, as described in the literature [25]. This variation in the hybrid materials (inorganic–organic) may be influenced by differences in temperature and time, as previously mentioned for the various synthesized samples. In addition, the values presented are consistent with variations in the plane (003), probably due to the exchange and orientation of the intercalated anions, and with the appearance of the plane (001), present in some intercalated samples, since the lamellar layers tend to be more significant in receiving the anions of INDO.

The band recorded in the LDH sample (Figure 2) has an acute peak at 1355 cm^−1^ due to the vibrational mode ν3 of NO_3_^−^ with D3h symmetry, suggesting the presence of nitrate anions [21]. In addition, a low-frequency vibration at 465 cm^−1^, present in both samples, characterizes the vibrational modes of binding between metals and oxygen, O-M-O, contained in the lamellar structure [26]. A band is also present at 1679 cm^−1^, characteristic of the benzoyl group (C=O), forming α, β-unsaturated ketones, in addition to 1548 cm^−1^ and 1478 cm^−1^, referring to the C=C aromatic rings of INDO [27]. In the intercalated sample, it is possible to observe an increase in band tendency in the region of 1324 to 1679 cm^−1^, corresponding to the asymmetric stretching carboxylate (COO-), characteristic of the sodium trihydrate form of INDO, confirming correlation and indicating drug intercalation in the anionic form, and conforming with other studies [28].

The morphology of these materials (Figure 3) reveals an LDH structure (Figure 3A,B) formed by disorganized agglomerates. However, at 16 h and 50 °C, LDH begins to present a plaque formation, probably related to the conditions of lamellar structural formations, as explained in the previous results of XRD, where the number of lamellae stacked is higher under these synthesis conditions. The intercalation micrograph at 16 h and 50 °C (Figure 3C) shows the formation of platelet-shaped agglomerates stacked as leaves, confirming the best lamellar structural formation. It is possible to observe the clear formation difference between the particles as a function of the synthesis time for the LDHs in Figure 3A,B; at 16 h and 50 °C, the LDHs presented are still in formation while, at 16 h and 90 °C, they consist of particles forming well-defined layers in the form of randomly stacked rounded platelets.

Thus, when correlating with the micrographs of LDHs, it is possible to observe that an increase in synthesis temperature directly influences the growth and morphological organization, as observed by Henrist [29]. However, for the samples intercalated for 16 h at 90 °C (Figure 3D), the micrographs show lower particle formation, as disorganized agglomerates of different sizes reveal heterogeneity in these interleaves. These results follow studies conducted by Theiss et al. in 2013 [30], in which LDH (Mg^2+^/Al^3+^) was synthesized with predominantly carbonate ions in the interlayer laminate. It is also essential to note minor variations in temperature in the thermal events associated with other studies, since the anion changes involved in the synthesis are correlated [22].

From TGA analysis, the INDO alone (Figure 4) showed two significant events at 200 °C, the first being a very high loss of mass of 84.9%, followed by a further 16.64% loss, via oxidation of the -OCH_3_ and -CH_2_COO^−^ functional groups, leading to the collapse of the structure at 600 °C [25,28,30]. For the intercalated samples, it is essential to note that the thermal decomposition steps may not be well-defined due to the overlap in the TGA curves. By comparing the isolated INDO with the intercalated sample, the TGA curves showed four significant mass losses ranging from 13.02% to 29.99% in the temperature range between 100 °C and 600 °C, coinciding with the temperature of the highest exothermic peak of the curve of differential thermal analysis (DTA) in Figure 5. Relating the GA to the DTA of Figure 5, it was impossible to observe any exothermic peak characteristic of crystalline phase formation, unlike in pure INDO and LDH, which can be observed in Figure 5. It is possible that the intercalated sample has better thermal stability than LDH and INDO alone because the influence of the drug present in LDH-INDO is equal to or slightly higher than that of INDO alone. Thus, the TGA compared to the DTA confirms that the intercalation presents a better thermal profile than the LDH or the pure INDO.

The calibration curve (Figure S2 and Tables S1–S3) of the UV-vis shows that there was a linear relationship between the absorbances and the indomethacin concentrations, and the linearity of the method was evaluated in the concentration range of 4 to 40 mg/L, since the curve has a correlation coefficient greater than 0.99. The results showed a good sensitivity of the method for quantification of indomethacin in the intercalated materials, evidencing the sample synthesized at 16 h and 50 °C as the one that presented the highest percentage of intercalation, with 86%.

Both INDO and INDO-LDH demonstrated more significant cytotoxicity for breast cancer cell lines (MDA-MB-231 and MCF-7). Using nuclear magnetic resonance spectroscopy, Natarajan, Glunde, and collaborators predicted that the treatment dose of these same cell lines with INDO would be between 0.05 mM and 0.3 mM [9,31]. The results of this study are, in part, consistent with those found by these authors, where the IC50 observed in MDA-MB-231 was 0.39 mM. The same authors suggested that the action of INDO on triple-negative breast cancer would be linked to the inhibition of COX and phosphocholine metabolism.

Furthermore, the MDA-MB-231 cell line was treated for 2 h with 0.3 mM INDO, and it was observed by microarray assay that a decrease in the expression of genes related to angiogenesis, apoptosis, cell adhesion, cell cycle regulation, cell differentiation, cell motility, choline phospholipid metabolism, and DNA repair occurred [32]. It was also observed that COX blockade by INDO leads to increased expression in the MCF-7 and MDA-MB-231 lines of interferon and chemokine receptors linked to tumor growth block, tumor immunity, and recruitment of natural killers. This increased expression profile would be linked to a better response to various chemotherapeutic regimens [33].

Terry and coworkers, in a case-control study with 1442 patients and 1420 controls, demonstrated a reduction in the risk of estrogen receptor-positive breast cancer with aspirin, finding a correlation between the use of NSAIDs and the non-development of this type of cancer [34]. It is important to remember that the MCF-7 line is positive estrogen receptor breast cancer, and MDA-MB-231 is a triple-negative breast tumor line, a subtype characterized by the absence of hormonal receptors and HER-2, for which there is no defined treatment protocol. For all the reasons already described in this study, INDO and LDH-INDO could be integrated as chemotherapeutic treatments. However, due to its low significant cytotoxicity to normal cells, despite the increase in IC50, using LDH-INDO would be preferable to using INDO by increasing the therapeutic window.

The same suggestion of treatment could be valid for patients with intestinal gastric cancer cell lines with the formation of ascites fluid (AGP01). The data resulting from this study show that the isolated form has an IC50 (0.51 mM) slightly lower than the MCF-7 line (0.52 mM). For LDH-INDO, the IC50 for AGP01 was 2.51 mM, which was even lower than for ACP02 and ACP03. Other than what has been noted for the other gastric tumor cell lines, this probably occurs since INDO inhibiting mechanisms lead to survival, cell migration, and metastasis in gastric cancer [35,36].

The results of the IC50 evaluation for the gastric cancer cell lines, specifically diffuse-type gastric cancer (ACP02) and intestinal type (ACP03), proved to be higher than the IC50 for MRC-5 and MN01. However, considering that the average IC50 for normal cells when exposed to LDH-INDO is 2.95 mM, we cannot rule out its use, with caution, as a coadjuvant in chemotherapy regimens for diffuse-type gastric cancer (IC50 = 2.85 mM). Previous studies have demonstrated that INDO, at a dose of 0.25 mM, begins to promote apoptosis of gastric cancer cells through the degradation of surviving and aurora-B proteins, related to resistance to apoptosis [36].

**Figure 7:**
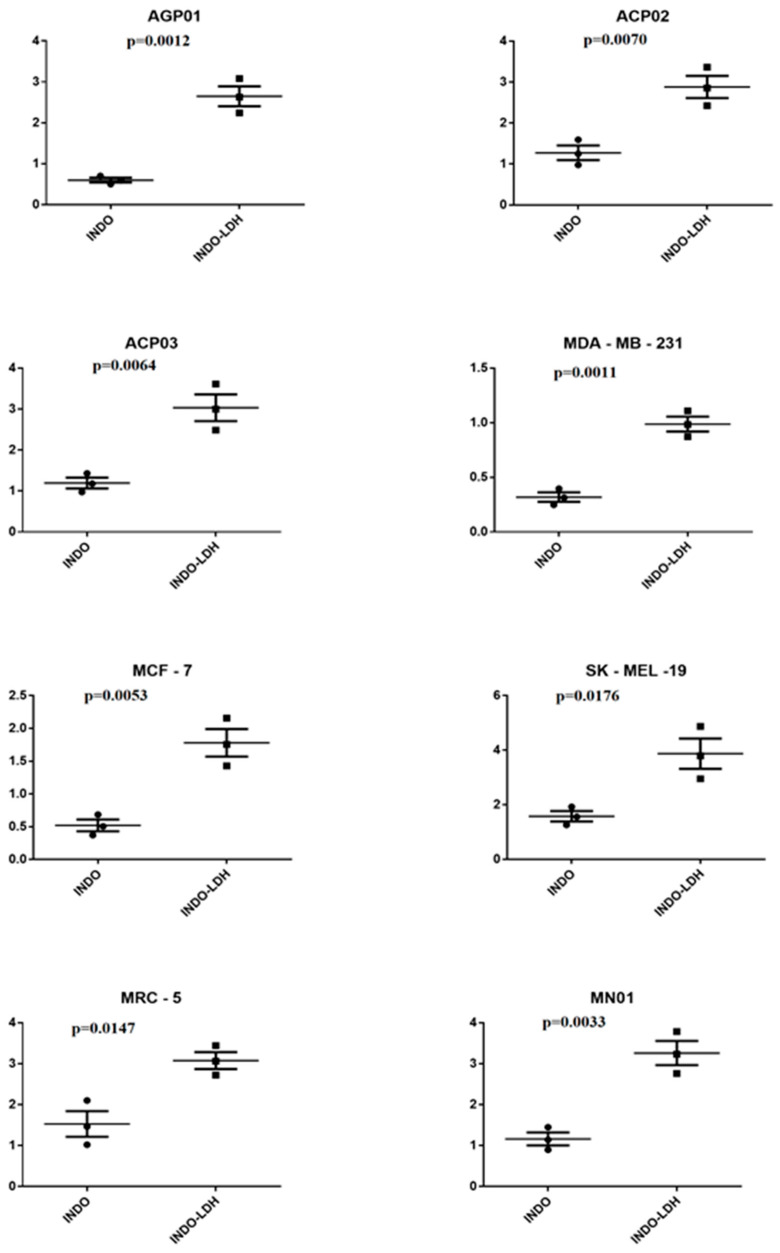
Non-paired Student’s *t*-test for the INDO group vs. LDH-INDO group, according to the indicated strain, with a significant difference of *p* < 0.05 for all strains. The confidence interval (CI) is indicated on the *Y*-axis.

In the literature, a type of melanoma known as “melanoma resistant to apoptosis induced by ligand tumor necrosis factor” (TRAIL-Melanoma) has been proposed to result from the use of isolation [37]. In this case, INDO would also act by degrading the SURVIVINE protein and activating cell death receptor 5. However, because the IC50 for melanoma with B-RAF mutation (represented by strain SK-MEL-19) is more significant than the IC50 for normal cells, neither INDO nor LDH-INDO would have the potential to be used in the treatment of this specific type of melanoma. A Student’s *t*-test was performed to verify if there was a statistically significant difference between the obtained data. The results were grouped into comparative groups, INDO vs. LDH-INDO and by individual lineage, with results presented in Figure 7.

**Figure 8:**
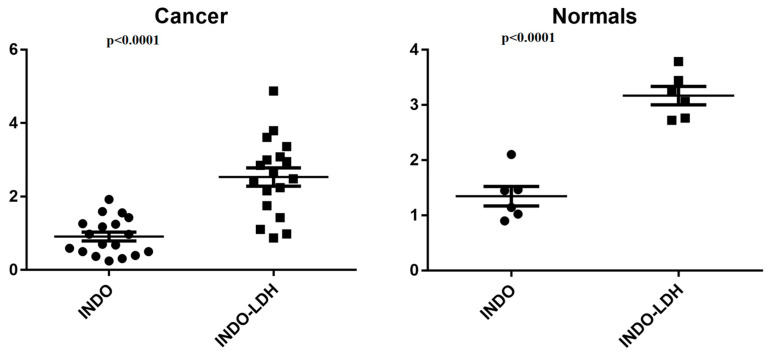
Student’s *t*-test, unpaired group, INDO vs. LDH-INDO lineages of the indicated cancer groups (AGP01, ACP02, ACP03, MCF-7, MDA-MB-231, and SK-MEL19) and clusters of regular cell groups (MRC-5 and MN01), with a significant difference (*p* < 0.0001).

**Figure 9:**
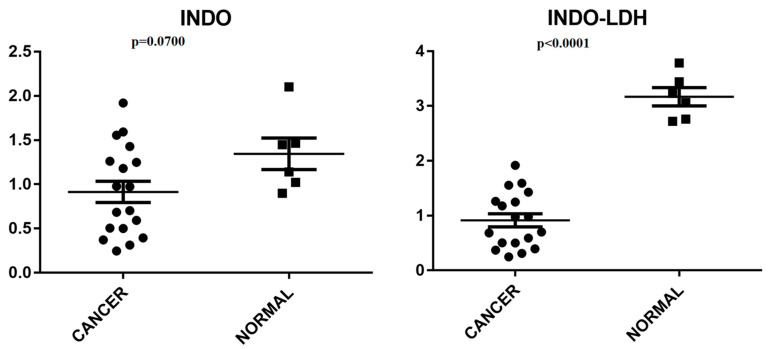
Student’s *t*-test, non-paired, for the INDO-treated group with cancer vs. non-neoplastic cell lines (Normals). Treatment of LDH-INDO lineages of cancer vs. non-neoplastic cell lines (Normals).

Figure 8 displays the pure INDO and LDH-INDO results in cancer cell lines. Meanwhile, Figure 9 illustrates the effects of INDO treatment in cancer cell lines compared to non-neoplastic cells and the impact of LDH-INDO treatment in both cancer and non-neoplastic cell lines. In the statistical analysis, a protective effect of the intercalation of indomethacin in the LDH matrix in both groups (*p* < 0.0001) was also observed by grouping cancer (Cancer) and non-neoplastic lines (Normals).

However, in the statistical analysis, comparing the treatment using INDO and LDH-INDO in clusters of cancer cells grouped vs. clusters of normal cells grouped, it was observed that only the treatment with LDH-INDO showed a statistically significant difference between the two groups, Cancer and Normal (*p* < 0.0001). The effect of INDO alone appeared to be the same for both cancer and normal cells. Thus, as a consequence, during a hypothetical treatment, the two cell types would suffer from the cytotoxicity of the drug, which, according to the results of this study, would not occur during a treatment using the INDO intercalated in the LDH matrix. Therefore, despite the increase in IC50, the use of LDH-INDO is preferable to the use of non-LDH-INDO.

## 4. Materials and Methods

### 4.1. Syntheses

The LDH samples were synthesized with Mg^2+^/Al^3+^ at a 2 molar ratio by coprecipitation at a constant pH ≈ 10, with some adaptations to optimize the method [28]. Two solutions were used for the synthetic route. First, solution A was prepared, containing 0.257 mol·L^−1^ de Mg(NO_3_)_2_·6H_2_O (Sigma-Aldrich, São Paulo, Brazil, 98%) and 0.128 mol·L^−1^ of Al(NO_3_)_3_·9H_2_O (Sigma-Aldrich, 98%), and then dissolved in decarbonated deionized water (H_2_O_dd_) using ELGA Purelab Option lab water (ELGA LabWater Brasil, São Paulo, Brazil). Subsequently, solution A was added slowly, under vigorous stirring, to solution B, containing 10 mL of 2 M sodium hydroxide (NaOH). The pH (~10) in the solution was kept constant by successive additions of NaOH. At the end of the addition, the mixture was maintained under vigorous stirring at a temperature of 50, 70, or 90 °C for 8, 16, 24, and 48 h.

All preparations took place under nitrogen atmosphere conditions to avoid contamination by CO_2_ and at a temperature below 303 K to prevent the formation of another phase, such as precipitation of simple hydroxides [38]. Subsequently, the suspension was centrifuged at 10,000 rpm for five minutes, and the decanted sample was gradually washed with H_2_O_dd_ and exposed to successive vacuum filtration procedures until the supernatant had a constant neutral pH value (~7.0). The resulting material was oven-dried at 60 °C for 24 h and then sprayed for further characterization.

LDH-INDO hybrids were synthesized at different proportions of LDH:INDO (2:1, 4:1, 6:1, and 8:1) using a similar methodology to what has been described above; however, some adaptations were added to improve the method used. Due to the presence of the drug on the route, solution A was prepared using 0.128 mol·L^−1^ Mg(NO_3_)_2_·6H_2_O (Sigma-Aldrich, 98%) and 0.064 mol·L^−1^ Al(NO_3_)_3_·9H_2_O (Sigma-Aldrich, 98%). The metal cations were also dissolved in H_2_O_dd_, so the predicted synthesis ratio was obeyed. In solution B, 2 g of INDO (Sigma-Aldrich, ≥99%) was diluted in a hydro-alcoholic solution (50% *v*/*v*). After that, the entire experimental protocol mentioned above was performed, following the description of LDH synthesis using the constant pH coprecipitation method.

### 4.2. Samples Characterization

The chemical composition, structure, and morphology of the samples were characterized by X-ray diffraction (XRD), scanning electron microscopy (SEM), and differential thermogravimetric analysis (TGA/DTA).

The XRD diffractograms were scanned in the range of 2θ 2–90° angle at a residence time of 2° every 22 s using a copper source with CuKα monochromatic radiation (λ = 1.5406 Å) and a D8 Advance Diffractometer from Bruker (Bruker do Brasil, Atibaia, Brazil) with a LynxEye PSD detector. The adjustments to the experimental parameters to obtain interplanar distances (dhkl), Muller indices (hkl), crystallite size (T), and network parameters (a and c) were performed using Peakoc software version 1.0 (https://www.esrf.fr, accessed on 1 January 2024).

FT-IR spectra were recorded on a Bruker Vertex 70 V spectrometer in the 400–4000 cm^−1^ range at 4 cm^−1^ resolution and across 64 scans. The micrographs for the study of SEM in LDH and LDH-INDO were obtained using a JEOL-JEM-6700 scanning electron microscope (JEOL Brazil, São Paulo, Brazil). Gold coverage was applied to the sample for conductivity at an acceleration voltage of 5.0 kV over 15 s with an electron beam current of 15 mA and a distance of 35 cm^−1^. Double-sided copper tapes were used to deposit the powder and adhere it to the aluminum sample port.

Thermogravimetric measurements were performed using an alumina crucible on the Shimadzu equipment (model DTG-60H) (Shimadzu Corporation, Barueri, Brazil). The measurement conditions were as follows: under nitrogen gas flow at a ratio of 70 mL·min^−1^, from 20 to 1000 °C, at a heating rate of 10 °C·min^−1^. The encapsulation efficacy was determined by the Shimadzu UV2600 Absorption Spectrophotometer (UV-VIS) (Shimadzu Corporation, Barueri, Brazil), measured by absorbance at λ = 320 nm, applying the Beer–Lambert law and using a linear equation obtained by linear regression from an analytical curve containing 10 points [39]. The solutions used for UV-vis analyses were prepared from the LDH-BMI samples diluted in 0.5 M hydrochloric acid (HCl, Sigma-Aldrich, 37%) + EtOH (CH_3_CH_2_OH, Sigma-Aldrich, 98%) under magnetic stirring for 20 min to dissolve the LDH-BMI, followed by subsequent successive dilutions with phosphate buffer at pH 7.4 [21].

### 4.3. Anticancer Activity

#### 4.3.1. Cellular Lines

Gastric adenocarcinoma cells obtained from patients with ascitic fluid were used for intestinal gastric adenocarcinoma (AGP01), (ACP02), and primary intestinal gastric adenocarcinoma tumor (ACP03), established at the Human Cytogenetic Laboratory (LCH) at the Biological Sciences Institute of the Federal University of Pará [40]. Mammary adenocarcinoma, estrogen receptor-positive (MCF-7), and triple-negative mammary adenocarcinoma (MDA-MB-231) cells were provided by Dr. Vanessa Morais Freitas (São Paulo University). Melanoma cells mutated in BRAF-serine/threonine B protein kinase (SK-MEL-19) were donated by Dr. Raquel Carvalho Montenegro from the Department of Physiology and Pharmacology within the Medicine Faculty at the Federal University of Ceará. Finally, lung fibroblast cells (MRC-5) were purchased from a governmental institution, the Cell Bank of Rio de Janeiro, and non-neoplastic gastric mucosal cells (MN01) were also established at the LCH.

#### 4.3.2. Cell Culture

Cells were grown under controlled conditions using appropriate culture bottles and media suitable for each lineage, were supplemented with 10% fetal bovine serum (FBS) and 1% of the antibiotics streptomycin and penicillin, and kept in an oven with 5% CO_2_ at 37 °C. Confluences were constantly monitored under an inverted microscope and the culture media were exchanged when necessary. The media were discarded following the experiments, and the cells were washed with Hank’s solution and trypsinized to detach them from the bottom of the bottle.

#### 4.3.3. MTT Cytotoxicity Assay

Cells were seeded in 96-well plates with 3 × 10^3^ cells per well and maintained in an oven with a 5% CO_2_ atmosphere at 37 °C. After waiting 24 h for adherence, the cells were treated with LDH-INDO (stock solution of 0.0302 g·mL^−1^) and INDO (stock solution of 0.0368 g·mL^−1^). From these stock solutions, serial triplicate dilutions were performed to obtain the curve from 0.0625 mM to 4 mM (100 μ/well), and a negative control that was not treated was established. After an incubation period of 72 h, the cell supernatant was aspirated and 100 μL of MTT solution (0.5 mg/mL in medium without bovine fetal serum (SBF)) was added. The plate was re-incubated in the oven at 5% CO_2_ for 3 h. After that, the liquid on top was removed and the plate was kept at 37 °C, shielded from light, for 24 h. Then, 100 μL of DMSO was added to each well, and the plate was stirred for 20 min until the formazan crystals were wholly dissolved. The plates were then analyzed using a plate spectrophotometer at a wavelength of 570 nm.

#### 4.3.4. Data Analysis

For data analysis, GraphPad Prism 5.0 software was used to create a non-linear regression of the percentage of inhibition versus the log of the concentrations, thus obtaining the inhibitory concentration with 50% of activity (IC50). A 95% confidence interval and a determination coefficient greater than 0.8 (R^2^ > 0.8) were allowed.

## 5. Conclusions

Here, XRD results show the direct influence of aging and temperature variations on LDH formation and indomethacin (INDO), prepared using the coprecipitation method at a constant pH of 10. The influence of these variables suggests the maximum interpolation limit of INDO at a 16 h time interval and a temperature of 50 °C. The micrographs showed agglomerated, rounded, randomly distributed platelet particles, mainly when synthesized at 90 °C. Thus, the characterization presented confirms that an increase in temperature and time of synthesis makes the material more organized; however, the higher the temperature increase and the time of synthesis, the larger the size of the crystals and order state of crystalline perfection, and this hinders the intercalation process, due to the high degree of organization of the lamellar structures.

In general, our results showed that the intercalation at 16 h and 50 °C was the sample that presented better structural organization and possibly more significant intercalation. These results were confirmed in vitro; interleaving a magnesium and aluminum hybrid matrix INDO decreased their cytotoxicity to normal cells, bringing the benefit of minimizing the side effects that could arise from its use in isolated form. In addition, INDO intercalated in the LDH matrix can be used both as a component and as a coadjuvant of the chemotherapy protocols for triple-negative breast cancer, estrogen receptor-positive breast cancer, gastric cancer with ascitic fluid formation, and diffuse-type gastric cancer, preferable to the use of unmanaged INDO. Functional studies will still need to be conducted to determine characteristics of drug action, such as cytotoxicity time, cell death mechanism, and action pathways. This study shows that relocating INDO through its LDH pathway presents new possibilities for cancer treatment.

## Figures and Tables

**Table 1:** Identification of the diffraction parameters of XRD of LDH samples.

Samples	Temperature (°C)	d_(003)_ (Å)	d_(110)_ (Å)	2θ (Å)	Particle Size T(*hkl*) [Å]	N° Stacking
LDH-8 h	50 °C	8.5404	1.5148	10.3495	79.2333	9.2773
LDH-8 h	70 °C	8.5166	1.5146	10.4156	96.4288	11.3224
LDH-8 h	90 °C	7.8816	1.5177	11.2173	186.4979	23.6622
LDH-16 h	50 °C	8.8025	1.5229	10.0406	196.9760	22.3771
LDH-16 h	70 °C	8.8027	1.5223	10.0404	206.9817	23.5134
LDH-16 h	90 °C	8.7963	1.5239	10.0477	177.4458	20.1726
LDH-24 h	50 °C	8.3150	1.5199	10.6309	93.6364	11.2611
LDH-24 h	70 °C	7.6017	1.5222	11.6317	596.7659	78.5037
LDH-24 h	90 °C	7.6507	1.5141	11.5571	392.5261	51.3059
LDH-48 h	50 °C	7.8247	1.5123	11.2992	169.0668	21.6067
LDH-48 h	70 °C	8.2095	1.5088	10.7679	538.4711	65.5906
LDH-48 h	90 °C	8.7205	1.5239	11.4521	597.7298	77.4205

**Table 2:** Identification of the diffraction parameters obtained from the XRD of the HDL-INDO samples.

Samples	Temperature (°C)	d_(001)_ (Å)	d_(003)_ (Å)	d_(110)_ (Å)	2θ (Å)	Particle Size T(*hkl*) [Å]	N° Stacking
LDH-INDO-8 h	50 °C	12.6614	8.4211	1.5154	10.4966	213.0154	25.2952
LDH-INDO-8 h	70 °C	25.2083	12.2255	1.5158	7.2249	300.9974	24.6204
LDH-INDO-8 h	90 °C	42.5754	12.0456	1.5085	7.3329	198.9354	16.5151
LDH-INDO-16 h	50 °C	42.5323	12.3747	1.5134	7.1377	135.1622	10.9224
LDH-INDO-16 h	70 °C	41.6789	11.2367	1.5154	7.2190	170.1067	4.0813
LDH-INDO-16 h	90 °C	40.8845	11.3459	1.5142	7.1276	162.8568	3.9833
LDH-INDO-24 h	50 °C	32.9748	12.2700	1.5115	7.1986	225.1180	18.3469
LDH-INDO-24 h	70 °C	20.4234	9.8009	1.5085	9.0156	210.8176	21.5100
LDH-INDO-24 h	90 °C	23.1638	13.5995	1.5120	6.4941	132.0095	9.7068
LDH-INDO-48 h	50 °C	26.1137	12.6181	1.5102	6.9998	836.2663	66.2746
LDH-INDO-48 h	70 °C	21.8045	10.8109	1.5116	8.1718	158.8192	14.6905
LDH-INDO-48 h	90 °C	25.7321	18.1079	1.5168	4.8761	175.9703	9.7178

**Table 3:** Network parameters of LDH and LDH-INDO samples.

Samples	Temperature (°C)	Network Parameters	
		a (Å)	c (nm)
LDH-8 h	50 °C	3.9896	0.2562
LDH-8 h	70 °C	3.0293	0.2554
LDH-8 h	90 °C	3.5354	0.2364
LDH-16 h	50 °C	3.0437	0.2653
LDH-16 h	70 °C	3.0447	0.2640
LDH-16 h	90 °C	3.0478	0.2638
LDH-24 h	50 °C	3.0398	0.2494
LDH-24 h	70 °C	3.0444	0.2280
LDH-24 h	90 °C	3.8282	0.2295
LDH-48 h	50 °C	3.8247	0.2347
LDH-48 h	70 °C	3.6177	0.2462
LDH-48 h	90 °C	3.0478	0.2316
LDH-INDO-8 h	50 °C	3.0309	0.3798
LDH-INDO-8 h	70 °C	3.0317	0.3667
LDH-INDO-8 h	90 °C	3.0170	0.3613
LDH-INDO-16 h	50 °C	3.8904	0.3713
LDH-INDO-16 h	70 °C	3.0309	0.3371
LDH-INDO-16 h	90 °C	3.0285	0.3403
LDH-INDO-24 h	50 °C	3.0844	0.3681
LDH-INDO-24 h	70 °C	3.0171	0.2940
LDH-INDO-24 h	90 °C	3.0240	0.2922
LDH-INDO-48 h	50 °C	3.0204	0.2518
LDH-INDO-48 h	70 °C	3.0232	0.3243
LDH-INDO-48 h	90 °C	3.0337	0.2540

**Table 4:** IC50 for INDO and LDH-INDO after 72 h of treatment.

Cell Lineage	INDO			LDH-INDO		
	IC_50_ (mM)	CI (95%)	R^2^	IC50 (mM) *	CI (95%)	R^2^
AGP01	0.51	0.46–0.56	0.98	2.51	2.05–3.07	0.80
ACP02	1.24	0.97–1.59	0.90	2.85	2.42–3.36	0.93
ACP03	1.18	0.97–1.42	0.93	2.99	2.48–3.61	0.92
MDA-MB-231	0.39	0.33–0.46	0.95	0.89	0.81–0.96	0.98
MCF-7	0.52	0.40–0.66	0.90	1.72	1.42–2.09	0.93
SK-MEL-19	1.55	1.26–1.92	0.91	3.79	2.95–4.87	0.84
MRC-5	0.99	0.85–1.15	0.95	2.68	2.34–3.08	0.93
MN01	1.14	0.89–1.44	0.90	3.23	2.76–3.78	0.92

* Non-linear regression was used to obtain the IC50, considering a confidence interval (CI) of 95%.

## Data Availability

The original contributions presented in the study are included in the article/Supplementary Material, further inquiries can be directed to the corresponding authors.

## Supplementary Materials

The following supporting information can be downloaded at: https://www.mdpi.com/article/10.3390/molecules29143353/s1, Figure S1. (A) Diffractograms and (B) samples deconvolution of HDL-16H. Figure S2. FT-IR plots for LDH-INDO, INDO and LDH systems at 50 °C and 16 h. Table S1. Results obtained in the INDO standard UV-Vis curve. Table S2. Intercalation Percent of INDO and HDL (1^a^ Reading). Table S3. Intercalation Percent of INDO and HDL (2^a^ Reading).

## References

[1] Sung H., Ferlay J., Siegel R.L., Laversanne M., Soerjomataram I., Jemal A., Bray F. (2021). Global Cancer Statistics 2020: GLOBOCAN Estimates of Incidence and Mortality Worldwide for 36 Cancers in 185 Countries. CA Cancer J. Clin..

[2] de Oliveira Santos M., de Lima F.C.D.S., Martins L.F.L., Oliveira J.F.P., de Almeida L.M., de Camargo Cancela M. (2023). Estimativa de Incidência de Câncer No Brasil, 2023–2025. Rev. Bras. Cancerol..

[3] Zhang K., Xu Z.P., Lu J., Tang Z.Y., Zhao H.J., Good D.A., Wei M.Q. (2014). Potential for Layered Double Hydroxides-Based, Innovative Drug Delivery Systems. Int. J. Mol. Sci..

[4] Barahuie F., Hussein M.Z., Fakurazi S., Zainal Z. (2014). Development of Drug Delivery Systems Based on Layered Hydroxides for Nanomedicine. Int. J. Mol. Sci..

[5] Rives V., del Arco M., Martín C. (2014). Intercalation of Drugs in Layered Double Hydroxides and Their Controlled Release: A Review. Appl. Clay Sci..

[6] Kim T.H., Lee G.J., Kang J.H., Kim H.J., Kim T.I., Oh J.M. (2014). Anticancer Drug-Incorporated Layered Double Hydroxide Nanohybrids and Their Enhanced Anticancer Therapeutic Efficacy in Combination Cancer Treatment. BioMed Res. Int..

[7] McCormick D.C., Edwards A.D., Wyatt J.S., Potter A., Cope M., Delpy D.T., Reynolds E.O.R. (1990). Effect of Indomethacin on Cerebral Oxidized Cytochrome-Aa3 Concentration in Preterm Infants. Pediatr. Res..

[8] Chennamaneni S., Zhong B., Lama R., Su B. (2012). COX Inhibitors Indomethacin and Sulindac Derivatives as Antiproliferative Agents: Synthesis, Biological Evaluation, and Mechanism Investigation. Eur. J. Med. Chem..

[9] Glunde K., Jie C., Bhujwalla Z.M. (2006). Mechanisms of Indomethacin-Induced Alterations in the Choline Phospholipid Metabolism of Breast Cancer Cells. Neoplasia.

[10] Agrawal A., Fentiman I.S. (2008). NSAIDs and Breast Cancer: A Possible Prevention and Treatment Strategy. Int. J. Clin. Pract..

[11] Boland G.P., Butt I.S., Prasad R., Knox W.F., Bundred N.J. (2004). COX-2 Expression Is Associated with an Aggressive Phenotype in Ductal Carcinoma in Situ. Br. J. Cancer.

[12] Mazhar D., Gillmore R., Waxman J. (2005). COX and Cancer. QJM.

[13] Jacobs J.W., Bijlsma J.W. (1997). NSAIDs: A Critical Appraisal. Neth. J. Med..

[14] Noreen F., Aman T., Mumtaz A., Nazir S. (2007). Spectrophotometric Determination of Indomethacin Using Partial Least Square Method. Proc. Pak. Acad. Sci..

[15] Tomisato W., Tanaka K.I., Katsu T., Kakuta H., Sasaki K., Tsutsumi S., Hoshino T., Aburaya M., Li D., Tsuchiya T. (2004). Membrane Permeabilization by Non-Steroidal Anti-Inflammatory Drugs. Biochem. Biophys. Res. Commun..

[16] Touhey S., O’Connor R., Plunkett S., Maguire A., Clynes M. (2002). Structure-Activity Relationship of Indomethacin Analogues for MRP-1, COX-1 and COX-2 Inhibition: Identification of Novel Chemotherapeutic Drug Resistance Modulators. Eur. J. Cancer.

[17] Ajmone-Cat M.A., Bernardo A., Greco A., Minghetti L. (2010). Non-Steroidal Anti-Inflammatory Drugs and Brain Inflammation: Effects on Microglial Functions. Pharmaceuticals.

[18] Ammoury N., Fessi H., Devissaguet J.P., Dubrasquet M., Benita S. (1991). Jejunal Absorption, Pharmacological Activi-ty, and Pharmacokinetic Evaluation of Indomethacin-Loaded Poly(d,l-Lactide) and Poly(Isobutyl-Cyanoacrylate) Nanocapsules in Rats. Pharm. Res..

[19] Srinath P., Diwan P.V. (1998). Pharmacodynamic and Pharmacokinetic Evaluation of Lipid Microspheres of Indomethacin. Pharm. Acta Helv..

[20] Srinath P., Vyas S.P., Diwan P.V. (2000). Preparation and Pharmacodynamic Evaluation of Liposomes of Indomethacin. Drug Dev. Ind. Pharm..

[21] Xu Z.P., Zeng H.C. (2001). Abrupt Structural Transformation in Hydrotalcite-like Compounds Mg_1−x_Al_x_(OH)_2_(NO_3_)_x_·nH_2_O as a Continuous Function of Nitrate Anions. J. Phys. Chem. B.

[22] Xu Z.P., Zeng H.C. (2001). Decomposition Pathways of Hydrotalcite-like Compounds Mg_1−x_Al_x_(OH)_2_(NO_3_)_x_·nH_2_O as a Continuous Function of Nitrate Anions. Chem. Mater..

[23] Mohanambe L., Vasudevan S. (2005). Anionic Clays Containing Anti-Inflammatory Drug Molecules: Comparison of Molecular Dynamics Simulation and Measurements. J. Phys. Chem. B.

[24] Rives V., Del Arco M., Martín C. (2013). Layered Double Hydroxides as Drug Carriers and for Controlled Release of Non-Steroidal Antiinflammatory Drugs (NSAIDs): A Review. J. Control. Release.

[25] Del Arco M., Cebadera E., Gutiérrez S., Martín C., Montero M.J., Rives V., Rocha J., Sevilla M.A. (2004). Mg,Al Layered Double Hydroxides with Intercalated Indomethacin: Synthesis, Characterization, and Pharmacological Study. J. Pharm. Sci..

[26] Aisawa S., Takahashi S., Ogasawara W., Umetsu Y., Narita E. (2001). Direct Intercalation of Amino Acids into Layered Double Hydroxides by Coprecipitation. J. Solid State Chem..

[27] Socrates G. (2004). Infrared and Raman Characteristic Group Frequencies.

[28] Mendieta S., Nuñez P.R., Oliva M., Pérez C., Fernández J., Crivello M. (2012). Intercalation of Anti-Inflammatory Drugs Sodium Indomethacin into Nanocomposites of Mg-Al. Structural Characterization. Procedia Mater. Sci..

[29] Henrist C., Mathieu J.P., Vogels C., Rulmont A., Cloots R. (2003). Morphological Study of Magnesium Hydroxide Nanoparticles Precipitated in Dilute Aqueous Solution. J. Cryst. Growth.

[30] Theiss F.L., Ayoko G.A., Frost R.L. (2013). Thermogravimetric Analysis of Selected Layered Double Hydroxides. J. Therm. Anal. Calorim..

[31] Natarajan K., Mori N., Artemov D., Bhujwalla Z.M. (2002). Exposure of Human Breast Cancer Cells to the Anti-Inflammatory Agent Indomethacin Alters Choline Phospholipid Metabolites and Nm23 Expression. Neoplasia.

[32] Ackerstaff E., Gimi B., Artemov D., Bhujwalla Z.M. (2007). Anti-Inflammatory Agent Indomethacin Reduces Invasion and Alters Metabolism in a Human Breast Cancer Cell Line. Neoplasia.

[33] Bronger H., Kraeft S., Schwarz-Boeger U., Cerny C., Stöckel A., Avril S., Kiechle M., Schmitt M. (2012). Modulation of CXCR3 Ligand Secretion by Prostaglandin E2 and Cyclooxygenase Inhibitors in Human Breast Cancer. Breast Cancer Res..

[34] Terry M.B., Gammon M.D., Teitelbaum S.L., Britton J.A., Neugut A.I. (2004). Association of Frequency and Duration with Breast Cancer Risk. JAMA.

[35] Guo Y.C., Chang C.M., Hsu W.L., Chiu S.J., Tsai Y.T., Chou Y.H., Hou M.F., Wang J.Y., Lee M.H., Tsai K.L. (2013). Indomethacin Inhibits Cancer Cell Migration via Attenuation of Cellular Calcium Mobilization. Molecules.

[36] Chiou S.K., Hoa N., Hodges A., Ge L., Jadus M.R. (2014). Indomethacin Promotes Apoptosis in Gastric Cancer Cells through Concomitant Degradation of Survivin and Aurora B Kinase Proteins. Apoptosis.

[37] Tse A.K.W., Cao H.H., Cheng C.Y., Kwan H.Y., Yu H., Fong W.F., Yu Z.L. (2014). Indomethacin Sensitizes TRAIL-Resistant Melanoma Cells to TRAIL-Induced Apoptosis through Ros-Mediated Upregulation of Death Receptor 5 and downregulation of Survivin. J. Investig. Dermatol..

[38] Costantino U., Ambrogi V., Nocchetti M., Perioli L. (2008). Hydrotalcite-like Compounds: Versatile Layered Hosts of Molecular Anions with Biological Activity. Microporous Mesoporous Mater..

[39] Miyata S. (1980). Physico-Chemical Properties of Synthetic Hydrotalcites in Relation to Composition. Clays Clay Miner..

[40] Leal M.F., Martins do Nascimento J.L., da Silva C.E.A., Vita Lamarão M.F., Calcagno D.Q., Khayat A.S., Assumpção P.P., Cabral I.R., de Arruda Cardoso Smith M., Burbano R.R. (2009). Establishment and Conventional Cytogenetic Characterization of Three Gastric Cancer Cell Lines. Cancer Genet. Cytogenet..

