# Supplementary material for: LDH-Indomethacin Nanoparticles Antitumoral Action: A Possible Coadjuvant Drug for Cancer Therapy
Source: Molecules. 2024 Jul 17;29(14):3353. doi: 10.3390/molecules29143353 (PMC11279815; doi:10.3390/molecules29143353)
Supplement: Supplementary file 1 [file molecules-29-03353-s001.zip › molecules-3071252-supplementary.pdf]

LDH-Indomethacin nanoparticles antitumoral action: A possible coadjuvant drug for cancer therapy

Kelly Costa Alves, Carlos Emmerson Ferreira da Costa, Cláudio Márcio Rocha Remédios, Danielle Queiroz Calcagno, Marcelo de Oliveira Lima, José Rogério A. Silva and Cláudio Nahum Alves

SUPPLEMENTARY MATERIAL

Figure S1. (A) Diffractograms and (B) samples deconvolution of HDL-16H.

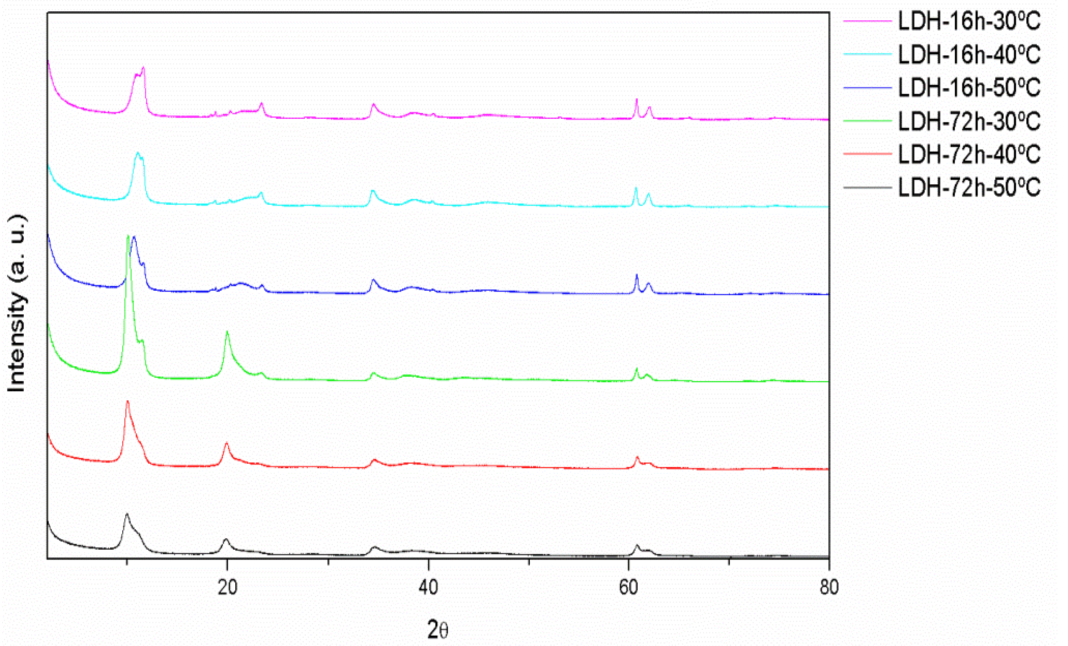

(A)

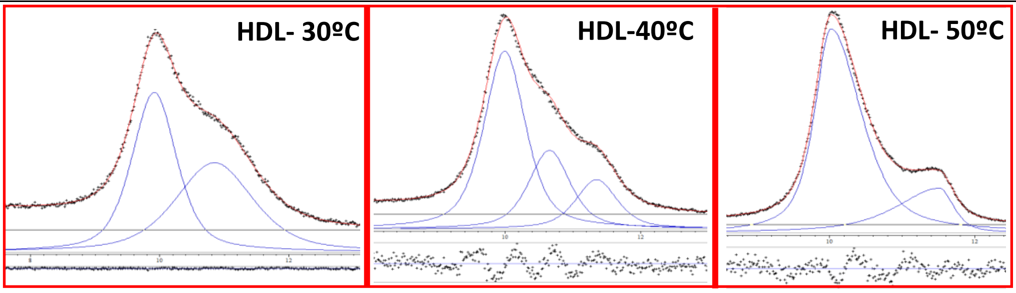

(B)

Figure S2. FT-IR plots for LDH-INDO, INDO and LDH systems at 50°C and 16h.

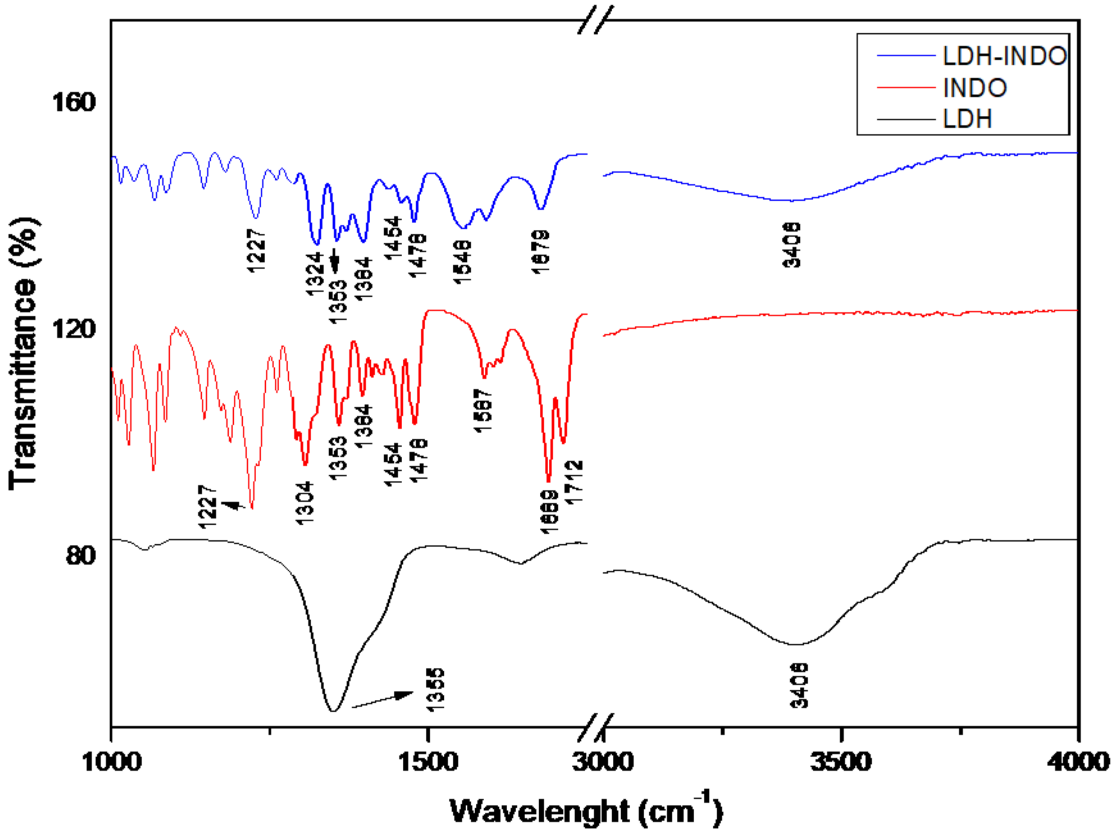

Figure S3. INDO standard UV-Vis curve.

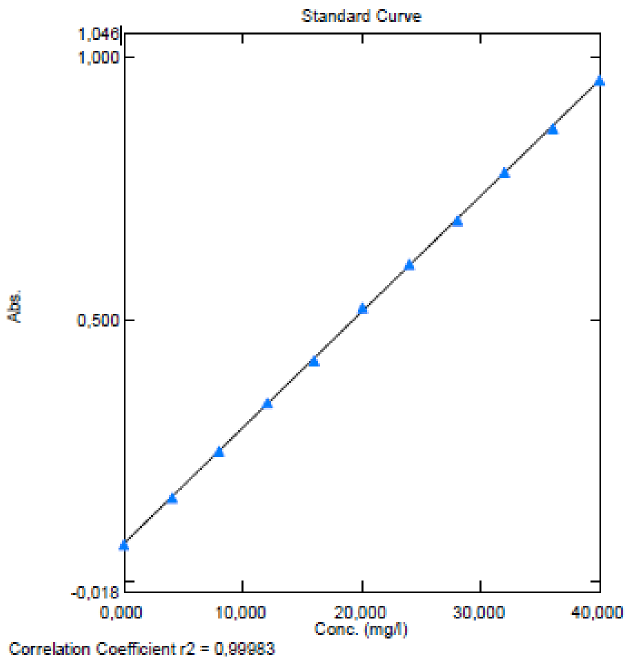

* The blue arrows are the absorbance points

** Correlation Coefficient r^2 = 0,99983 e Correlation Factor = 1

*** Straight Equation: y = 0,02891X + 0,04270 , being X=concentration e y=absorbance

Table S1. Results obtained in the INDO standard UV-Vis curve

**Solution**	**Concentration (mg.L^{-1})**	**Absorbance**			
		**1ª reading**	**2ª reading**	**3ª reading**	**Mean**
Blank	0	0.071	0.129	0.070	0.090
1	4	0.162	0.172	0.169	0.168
2	8	0.249	0.218	0.253	0.240
3	12	0.343	0.263	0.342	0.316
4	16	0.423	0.308	0.420	0.384
5	20	0.523	0.348	0.537	0.469
6	24	0.606	0.402	0.606	0.538
7	28	0.69	0.462	0.711	0.621
8	32	0.782	0.498	0.803	0.694
9	36	0.863	0.567	0.855	0.762
10	40	0.957	0.594	0.974	0.842

Table S2. Intercalation Percent of INDO and HDL (1ª Reading).

ID	Ratio (HDL:INDO)	% INDO ($mg.L^{-1}$) 50ºC	% INDO ($mg.L^{-1}$) 70ºC	% INDO ($mg.L^{-1}$) 90ºC
HDL – 8h	-	10.685	13.091	9.209
HDL – 16h	-	0.085	0.611	0.626
HDL – 24h	-	10.389	12.127	8.808
HDL – 48h	-	11.527	11.099	11.369
HDL – 72h	-	0.371	1.495	1.639
HDL – INDO- 8h	2:1	71.662	66.171	69.547
HDL – INDO- 16h	2:1	86.361	55.725	21.149
HDL – INDO- 24h	2:1	82.862	45.022	42.347
HDL – INDO- 48h	2:1	66.765	44.845	19.240
HDL – INDO-72h	2:1	38.321	39.661	34.097
HDL – INDO- 72h	4:1	49.970	6.006	7.130
HDL – INDO- 72h	6:1	16.648	3.922	4.229
HDL – INDO- 72h	8:1	3.829	3.681	11.018

*The ratio was determined by setting the indomethacin concentration and varying the HDL concentration.

Table S3. Intercalation Percent of INDO and HDL (2ª Reading).

ID	Ratio (HDL:INDO)	% INDO ($mg.L^{-1}$) 50ºC	% INDO ($mg.L^{-1}$) 70ºC	% INDO ($mg.L^{-1}$) 90ºC
HDL – 8h	-	10.707	12.079	9.242
HDL – 16h	-	0.085	0.611	0.626
HDL – 24h	-	10.284	12.148	8.701
HDL – 48h	-	11.850	9.826	11.494
HDL – 72h	-	0.371	1.495	1.639
HDL – INDO- 8h	2:1	71.561	66.350	66.829
HDL – INDO- 16h	2:1	86.269	55.561	21.093
HDL – INDO- 24h	2:1	82.562	45.698	42.409
HDL – INDO- 48h	2:1	67.454	44.784	19.299
HDL – INDO-72h	2:1	38.321	39.661	34.097
HDL – INDO- 72h	4:1	49.970	5.934	7.130
HDL – INDO- 72h	6:1	16.992	3.922	4.229
HDL – INDO- 72h	8:1	3.829	3.681	11.018

* The ratio was determined by setting the indomethacin concentration and varying the HDL concentration.
